# Retraction Note: Energy efficient IRS assisted 6G network for Industry 5.0

**DOI:** 10.1038/s41598-024-52445-1

**Published:** 2024-01-30

**Authors:** Ashu Taneja, Shalli Rani, Saleem Raza, Amar Jain, Shebnam M. Sefat

**Affiliations:** 1 Chitkara University Institute of Engineering and Technology, Chitkara University, Rajpura, Punjab 140401 India; 2 Quaid-E-Awam University of Engineering, Science and Technology, Larkana, Pakistan; 3 Symbiosis International University, Pune, India; 4 Gamo Gofa, Ethiopia

Retraction of: *Scientific Reports* 10.1038/s41598-023-39974-x, published online 07 August 2023

The Editors have retracted this Article.

An investigation conducted after its publication found evidence to suggest that the veracity of the authorship in this article cannot be ascertained.

Shebnam M. Sefat agrees with the retraction and its wording. Ashu Taneja, Shalli Rani, Saleem Raza, and Amar Jain disagree with the retraction.

After the publication of this Retraction Notice, the Arba Minch Institute of Technology informed the Editors that Shebnam M. Sefat is not and has never been affiliated with this institution. The Editors contacted Shebnam M. Sefat but could not determine their institutional affiliation at the time of the research. The Article and Retraction Note were updated to reflect this.

